# P202: Investigation of a cluster of invasive mold infections in a large teaching hospital

**DOI:** 10.1186/2047-2994-2-S1-P202

**Published:** 2013-06-20

**Authors:** A Gayet-Ageron, N Farquet, E Von Dach, C van Delden, V Camus, Y Chalandon, D Pittet, A Iten

**Affiliations:** 1 Infection Control Program, University Hospitals of Geneva, Geneva, Switzerland; 2 Department of Surgery, Transplant service, University Hospitals of Geneva, Geneva, Switzerland; 3 Service of Haematology, University Hospitals of Geneva, Geneva, Switzerland

## Introduction

In July 2010, we observed an increased number of invasive mold infections (IMI) in the hematology wards of our institution.

## Objectives

We assessed risk factors associated with IMI in order to optimize preventive and protective measures.

## Methods

Retrospective matched case-control study conducted in a 1900-bed teaching hospital between September 2008 and March 2011. Cases were defined as proven, probable, or possible according to standardized international consensus definitions. Controls were at-risk patients (onco-hematologic patients and allogeneic hematopoietic stem cell transplant recipients) hospitalized during the same time period as cases. Data were recorded by three investigators unblinded to the case-control status using a standardized case-report form. Conditional logistic regression was applied.

## Results

Between November 2008 and March 2011, a total of 29 cases were identified: 6 proven (20.7%), 8 probable (27.6%) and 15 possible cases (51.7%); 102 controls were matched to cases. Cases had a longer hospital stay (P<0.001) and were exposed to a longer duration of neutropenia (P<0.001). They differed from controls regarding the hospitalization ward (P=0.001), chemotherapy use in the prior year (P=0.002), the existence of prior cytomegalovirus (CMV) infection (P=0.03), and a higher number of examinations outside the ward before IMI diagnosis (P<0.001). By multivariate analysis, after adjustment for age, hospitalization ward, duration of neutropenia, and history of CMV infection, two independent factors were associated with IMI: length of hospital stay in days (OR 1.06, P=0.02) and the number of examinations outside the ward (OR 1.47; P=0.02).

## Conclusion

Our results suggest that cases were more exposed to environmental fungi and specific recommendations related to patient transport within the hospital were reinforced.

## Disclosure of interest

None declared

